# Enhancing urinary tract infection diagnosis for negative culture patients with metagenomic next-generation sequencing (mNGS)

**DOI:** 10.3389/fcimb.2023.1119020

**Published:** 2023-03-03

**Authors:** Kaipeng Jia, Shiwang Huang, Chong Shen, Hongjun Li, Zhe Zhang, Lei Wang, Gangjian Zhao, Zhouliang Wu, Yuda Lin, Han Xia, Mingze Tang, Huifen Yang, Hailong Hu

**Affiliations:** ^1^ Department of Urology, The Second Hospital of Tianjin Medical University, Tianjin, China; ^2^ Tianjin Key Laboratory of Urology, Tianjin Institute of Urology, The Second Hospital of Tianjin Medical University, Tianjin, China; ^3^ Department of Infectious Diseases, The Second Hospital of Tianjin Medical University, Tianjin, China; ^4^ Department of Scientific Affairs, Hugobiotech Co., Ltd., Beijing, China

**Keywords:** metagenomic next-generation sequencing (mNGS), diagnosis, urinary tract infection (UTI), treatment, high throughput sequencing (HTS)

## Abstract

**Background:**

Metagenomic next-generation sequencing (mNGS) is a promising technology that allows unbiased pathogen detection and is increasingly being used for clinical diagnoses. However, its application in urinary tract infection (UTI) is still scarce.

**Methods:**

The medical records of 33 patients with suspected UTI who were admitted to the Second Hospital of Tianjin Medical University from March 2021 to July 2022 and received urine mNGS were retrospectively analyzed. The performance of mNGS and conventional urine culture in diagnosing infection and identifying causative organisms was compared, and the treatment effects were evaluated in terms of changes in urinalyses and urinary symptoms.

**Results:**

In the detection of bacteria and fungi, mNGS detected at least one pathogen in 29 (87.9%) cases, including 19 (57.6%) with positive mNGS but negative culture results and 10 (30.3%) with both mNGS and culture positive results. The remaining 4 (12.1%) patients were negative by both tests. Overall, mNGS performed better than culture (87.9% vs. 30.3%, *P* < 0.001). Within the 10 double-positive patients, mNGS matched culture results exactly in 5 cases, partially in 4 cases, and not at all in 1 case. In addition, mNGS detected a broader pathogen spectrum, detecting 26 species compared to only 5 species found in culture. The most abundant bacteria detected by mNGS was *Escherichia coli*, detected in 9 (27.2%) patients. All anaerobic bacteria, *Mycobacterium Tuberculosis* and all mixed pathogens were detected by mNGS. The final clinical diagnosis of UTI was made in 25 cases, and the sensitivity of mNGS was significantly higher than culture (100.0% vs 40.0%; *P* < 0.001) when using the diagnosis as a reference standard; the positive predictive value, negative predictive value and specificity were 86.2%, 100% and 50.0%, respectively. Importantly, targeted antibiotic therapy based on mNGS resulted in significant improvement in urinalyses and urinary symptoms in patients.

**Conclusions:**

mNGS is a technology that has shown clear advantages over culture, particularly in the context of mixed infections and UTIs that are difficult to diagnose and treat. It helps to improve the detection of pathogens, guide changes in treatment strategies, and is an effective complement to urine culture.

## Introduction

Urinary tract infection (UTI) is one of the most common infections, affecting 150 million people worldwide each year. The most common cause of UTI is pathogens (mainly *Escherichia coli*) in the stool that ascend along the urinary tract and infect the bladder (Flores-Mireles et al., 2015). Pathogenic culture is the gold standard for the diagnosis of UTI. However, cultures are often time-consuming, with low detection rates and limited diagnostic accuracy, especially in patients who have been on antibiotics (Simner et al., 2018). Clinically, the patients are usually considered to be infected if they have white blood cells in high power field (WBCHPF) count > 5 in the urine and symptoms of urinary irritation such as frequency, urgency, and dysuria. Meanwhile empirical broad-spectrum antibiotic therapy is given in most cases, even in the absence of an identified pathogen. In reality, there is often a proportion of patients who continue to have chronic urinary irritation symptoms and abnormal urinalysis results that interfere with normal life, even after the above treatment. The burden of disease is further exacerbated by a dramatic increase in resistance of pathogenic bacteria due to severe antibiotic abuse (Flores-Mireles et al., 2015). Failure to obtain an accurate and timely diagnosis may lead to inappropriate treatment, more widespread antibiotic resistance, and increased costs. Although a variety of pathogen diagnostic methods are available, the choice of these diagnostic methods often requires prior judgment of clinician (Chu and Lowder, 2018; Han et al., 2019). Therefore, rapid and accurate identification of pathogens, professional interpretation and targeted treatment are key to clinical diagnosis and treatment of UTI (Li et al., 2020).

Advances in genome sequencing technologies and bioinformatics methods have provided favorable tools for the diagnosis of clinical infectious diseases, such as high throughput sequencing (HTS) (Hilton et al., 2016). Metagenomic next-generation sequencing (mNGS) is an emerging pathogen detection technology, capable of identifying all microbial genomic sequences in clinical specimens within 24 hours, and is an unbiased method that can theoretically detect any microorganism, especially in the detection of *Mycobacterium tuberculosis*, fungi, anaerobes, and viruses (Goldberg et al., 2015), thereby identifying rare, hard-to-detect, or mixed infection. Meanwhile this technique does not require clinical advance prediction(Miao et al., 2018). It is less influenced by prior antibiotic exposure (Miao et al., 2018), and can be used to predict antibiotic resistance and guide antimicrobial drug use through resistance gene testing (Han et al., 2019). This technology further facilitates accurate diagnosis and optimal treatment.

In this study, we applied the mNGS detection technology to UTI, compared it with conventional laboratory tests, and assessed the clinical outcomes of patients.

## Materials and methods

### Patient enrollment and general information

The medical records of 33 patients with suspected UTI admitted to the Second Hospital of Tianjin Medical University (Tianjin, China) from March 2021 to July 2022 who underwent urine mNGS testing were retrospectively analyzed. These patients had negative urine culture results prior to mNGS detection, but they had multiple abnormal urinalysis results and persistent urinary tract symptoms and were still suspected of having a UTI.

Most patients underwent the detection when they had poor responses to empirical antibiotic treatment, and a few patients underwent the detection directly without receiving empirical anti-infective treatment. All of them underwent a concomitant urine culture for comparison with mNGS in the diagnosis of infection and determination of the causative organism. Urinalysis using urine sediment analyzer and voiding symptoms were evaluated before and after anti-infective therapy.

### Specimen collection

A standard urine collection procedure was performed to obtain a minimum of 80ml of urine, with 3ml being utilized for mNGS examination, at least 20 ml for urinalysis, and 50 ml or more for urine culture. The results of the urine culture were then compared to those of the mNGS examination in order to evaluate any differences in pathogen detection. Patients were instructed to collect clean midstream urine after cleaning the area around the urethra. For patients who need to collect urine from a catheter or nephrostomy tube, the proximal sampling site of the catheter was cleaned and a syringe needle was inserted into the catheter lumen to aspirate urine after clamping the drainage tube for no more than 30 minutes. A total of 33 samples were collected in this study. Urinalyses were performed within 2 hours of collection; urine culture samples were sent to the microbiology laboratory at the Second Hospital of Tianjin Medical University within 2 hours of collection and processed according to recommended microbiological methods. mNGS samples were stored at 4 degrees Celsius after collection. All the patients signed an informed consent form.

### Isolation and detection of pathogens by culture

The culture, isolation and strain identification of bacteria and fungi from midstream clean urine specimens were completed according to the recommended microbiological methods. The quality control strains of Gram-positive cocci, Gram-negative bacilli and fungi were *Enterococcus casseliflavu* ATCC700327, *Enterobacter cloacae* ATCC700323 and *Candida krusei* ATCC 6258, respectively.

### Detection of pathogens by mNGS

After sample collection, samples were sent to the testing center within 24 hours on dry ice for PACEseq mNGS (Hugobiotech Co., Ltd., Beijing, China). Firstly, the cells in samples were removed by centrifugation, and the supernatant was collected for the subsequent DNA extraction. DNA extraction was performed using the QIAamp DNA Micro Kit (QIAGEN). Secondly, a DNA library construction was performed on the obtained extracts using the QIAseq™ Ultralow Input Library Kit (Illumina). The constructed libraries were assessed for quality using a Qubit fluorescence quantification analyzer (Thermo Fisher) and an Agilent 2100 Bioanalyzer (Agilent Technologies). The quality-qualified databases were subjected to macro-sequencing using the Nextseq 550 platform. Then, the low-quality, low-complexity and small fragment DNA sequences were removed from the raw files after mNGS sequencing; the human sequences matching to the human reference genome (hg38) were then screened out using BWA software. Finally, the remaining sequences were matched to the microbial reference genome database (ftp://ftp.ncbi.nlm.nih.gov/genomes/) (Li and Durbin, 2009). The species information of the genomic targets matched by the above sequences was retrieved, and the specific sequencing reads of all microorganisms and their ratio to the total number of sequences, i.e., relative abundance, were retrieved. In addition, negative controls (sterile deionized water) and positive controls (synthesize fragments with known quantities) were established for each batch of experiments using the same wet lab procedures and bioinformatics analysis as the clinical samples.

Ultimately, the report of mNGS detection provided the species, number of the specific sequencing reads, confidence level of all detected microorganisms (bacteria, fungi, viruses) as well as drug resistance gene testing.

### Making the final clinical diagnosis

After the results of culture and mNGS detection were returned, at least 3 urology, infectious disease and microbiology specialists worked together to interpret the reports, taking into account the patient’s clinical background, concomitant symptoms, and other microbiological and laboratory findings. They assessed whether the patient has a UTI and gave a final clinical diagnosis, and then jointly decided on a treatment plan.

### Assessment of urinary symptoms

Urinary symptoms before and after medication were assessed using overactive bladder syndrome score (OABSS). Total score (score: 0-15) and individual scores for daytime frequency (score: 0-2), nighttime frequency (score: 0-3), urgency (score: 0-5), and urgency incontinence (score: 0-5) were assessed. A total score of 3-5 was considered as mild voiding symptoms, 6-11 as moderate, and ≥12 as severe (**Supplementary Table 1**) (Homma et al., 2006). In addition, patients’ frequency of dyspareunia was assessed as: not at all, a few times, almost always, fairly often and usually (O’leary et al., 1997).

### Data analysis methods

The collected medical records were analyzed descriptively. A 2 × 2 column table was established to determine the sensitivity, specificity, positive predictive value (PPV) and negative predictive value (NPV) using the clinical diagnosis as the criteria. Statistical analysis was performed using SPSS (version 25.0) software, and data were plotted using Prism (version 8.0) software. Detection rates of mNGS were compared with conventional culture using McNemar’s test; paired t-test or Wilcoxon signed rank test was used to test paired data. *P* < 0.05 was considered statistically significant, and all tests were two-tailed. Bonferroni correction is applied to address the issue of multiple testing.

## Result

### Patient characteristics

Of the 33 patients enrolled, 22 were males and 11were females, with a mean age of 65.0 ± 12.0 years. Their demographic and clinical characteristics are summarized in **Table 1**. 22 of these 33 patients were tested after a poor response to empirical anti-infective therapy, and the remaining 11 patients chose to be detected without empirical antibiotic therapy. The included cases were all patients with clinically suspected UTI, and 25 were ultimately clinically diagnosed with UTIs.

**Table 1:** Demographic and clinical characteristics of the 33 patients.

Clinical characteristics	Results
Age (yr, mean ± SD)	65.0 ± 12.0
Gender, n (%)	
Male	22 (66.7%)
Female	11 (33.3%)
Empirical anti-infective treatment before mNGS testing, n (%)	
Yes	22 (66.7%)
No	11 (33.3%)
Voiding pattern, n (%)	
By urinary catheter	5 (15.2%)
By nephrostomy tube Vesicovaginal fistula	3 (9.1%) 1 (3%)
Voiding through the urethra	24 (72.7%)
WBC in the urinalysis, n (%)	
Normal	0 (0%)
Abnormal	33 (100%)
Irritation sign of bladder in the patients voiding through the urethra, n (%)	
Yes	24 (100%)
No	0 (0%)
Final clinical diagnosis of UTI, n (%)	
Present	25 (75.8%)
Absent	8 (24.2%)

mNGS, metagenomic next-generation sequencing; WBC, white blood cells; UTI, urinary tract infection.

### Results of the two methods

**Figure 1:**
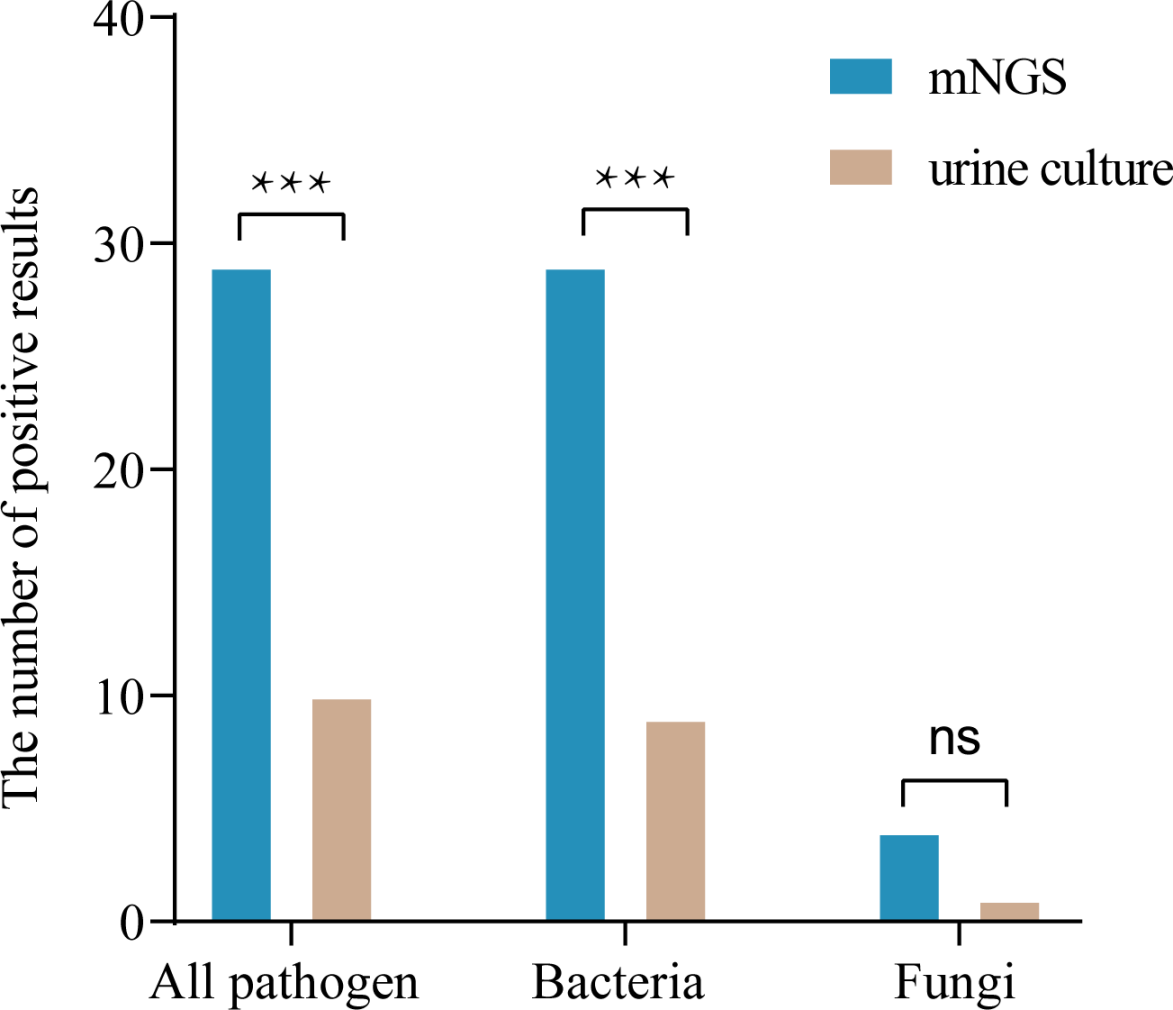
Comparison of diagnostic performance in pathogen detection between mNGS and culture (n=33). ***P < 0.001; ns, not significant.

Among the 33 patients, mNGS results were compared with conventional culture results in terms of detection of bacteria and fungi as shown in **Table 2** and **Supplementary Table 2**. The results of both methods were positive in 10 cases; of the 23 patients with negative urine culture results, only 4 were negative for mNGS, leaving 19 cases with at least one pathogen detected by mNGS; there were no cases with positive results by conventional culture and negative results by mNGS (**Table 2** and **Supplementary Table 2**). mNGS detected at least one pathogen in 29 (87.9%) urine samples, while urine culture detected only 10 (30.3%) cases (87.9% vs. 30.3%, P < 0.001). Compared with the urine cultures, mNGS detected more bacteria (87.9% vs. 27.3%, *P* < 0.001) and fungi (12.1% vs. 3.0%, *P* = 0.250). (**Table 3** and **Figure 1**).

**Table 2:** Comparison of mNGS and culture results in the UTI-suspected group (n=33).

mNGS	culture		all
	pos	neg	
pos	10	19	29
neg	0	4	4
all	10	23	33

**Table 3:** Comparison of diagnostic performance in pathogen detection between mNGS and culture (n=33).

Group	mNGS No. (%)	Urine culture No. (%)	P
All pathegen	29 (87.9)	10 (30.3)	<0.001
Bacteria	29 (87.9)	9 (27.3)	<0.001
Fungi	4 (12.1)	1 (3.0)	0.250

The sensitivity and specificity of mNGS for the diagnosis of UTI, were 100% and 50%; 86.2% and 100% for PPV and NPV, respectively. The sensitivity and specificity of conventional diagnostic methods for the diagnosis of infection were 40% and 100%, and 100% and 34.8% for PPV and NPV, respectively (**Table 4**). And the sensitivity of mNGS for the diagnosis of UTI was significantly higher than that of culture (100.0% vs 40.0%; *P* < 0.001).

**Table 4:** Performance of mNGS and conventional culture in diagnosis of UTI.

Diagnostic testing	sensitivity (%) (95% CI)	specificity (%) (95% CI)	PPV (%) (95% CI)	NPV (%) (95% CI)
mNGS	100.0 (86.3-100)	50.0 (15.7-84.3)	86.2 (68.3-96.1)	100.0 (39.8-100)
culture	40.0 (21.1-61.3)	100.0 (63.1-100)	100.0 (69.2-100)	34.8 (16.4-57.3)

PPV, positive predictive value; NPV, negative predictive value; CI, confidence interval.

### Results of pathogen detection

**Figure 2:**
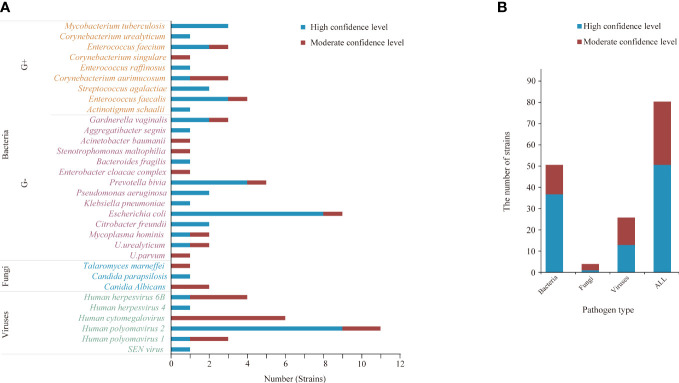
The microorganisms detected by mNGS. **(A, B)** A total of 81 strains of microorganisms were detected, including 51 bacteria, 4 fungi and 26 viruses, of which 51 were high confidence levels. **(B)** mNGS detected 23 species of bacteria, 3 species of fungi, and 6 species of viruses.

**Figure 3:**
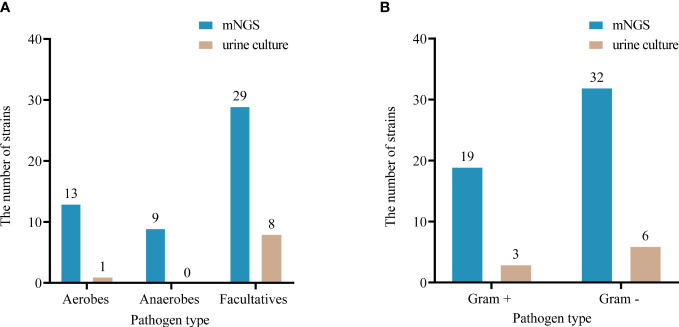
Comparison of oxygen requirements **(A)** and Gram staining characteristics **(B)** of bacteria detected by urine culture and mNGS.

Among the microorganisms (n=81) isolated by mNGS from 33 specimens, 51 were determined to be of high confidence and 30 of moderate confidence. There were 51 strains of bacteria, of which 37.3% (19/51) were Gram-positive and 62.7% (32/51) were negative; 4 strains of fungi and 26 strains of viruses (**Figures 2**, **3**). A total of 9 bacteria and 1 fungus were detected by culture. In terms of the detection of bacteria, both assays showed that the Gram-negative bacteria and facultative anaerobes were the most common (**Figure 3**). The most common bacteria detected by mNGS was *E. coli*, followed by *Prevotella* and *Enterococcus faecalis*; the most common bacteria detected by culture was also *E. coli*. Among the 33 specimens, mNGS detected 23 species of bacteria, 3 species of fungi, and 6 species of viruses. Out of these, 19 bacterial and 2 fungal species were undetected through conventional culture techniques. In addition, all viruses, *M. tuberculosis* and anaerobes were detected by mNGS (**Figures 2**, **3**). The mNGS performed significantly better than culture the detection of pathogens.

Among the 33 cases of mNGS detection, 22 patients were tested when their disease did not improve significantly or even worsened after treatment with empirical antibiotics, and 11 patients underwent testing directly. An overview of cultures and mNGS results in detection of bacteria and fungi in the two groups was demonstrated in **Table 5**. mNGS was not affected by previous use of antibiotics in detecting pathogens (90.9% VS. 81.8%, *P* = 0.586).

**Table 5:** Overview of traditional cultures and mNGS results in detection of bacteria and fungi.

	Total (n=33)	Anti (n=22)	Non (n=11)
Culture results			
Patients with positive results, n (%)	10 (30.3)	6 (27.3)	4 (36.4)
Number of detected pathogens			
All, n	10	6	4
Bacteria, n	9	5	4
Fungi, n	1	1	0
Gram stain			
Gram-positive bacteria, n	3	2	1
Gram-negative bacteria, n	6	3	3
Oxygen requirements			
Aerobes, n	1	0	1
Anaerobes, n	0	0	0
Facultative, n	8	5	3
5 most prevalent species, n (%)			
*Escherichia coli*	5 (15.2)	3 (13.6)	2 (18.2)
*Enterococcus faecium*	2 (6.1)	1 (4.5)	1 (9.1)
*Pseudomonas aeruginosa*	1(3.0)	0 (0)	1 (9.1)
*Streptococcus agalactiae*	1(3.0)	1(4.5)	0 (0)
*Candida tropicalis*	1(3.0)	1 (4.5)	0 (0)
mNGS results			
Patients with positive results, n (%)	29 (87.9)	20 (90.9)	9 (81.8)
Number of detected pathogens			
All, n	55	36	19
Bacteria, n	51	34	17
Fungi, n	4	2	2
Gram stain			
Gram-positive bacteria, n	19	15	4
Gram-negative bacteria, n	32	19	13
Oxygen requirements			
Aerobes, n	13	8	5
Anaerobes, n	9	7	2
Facultative, n	29	19	10
5 most prevalent species, n (%)			
*Escherichia coli*	9 (27.3)	5 (22.7)	4 (36.4)
*Prevotella bivia*	5 (15.2)	4 (18.2)	1 (9.1)
*Enterococcus faecalis*	4 (12.1)	4 (18.2)	0 (0)
*Gardnerella vaginalis*	3 (9.1)	2 (9.1)	1 (9.1)
*Enterococcus faecium*	3 (9.1)	2 (9.1)	1 (9.1)

The results in two groups of suspected UTI patients who had previously received (anti) and did not (non) receive empirical anti-infective therapy.

Of these 33 patients, 24 patients voided through the urethra and 8 patients voided through the catheter (including urinary catheters and nephrostomy tubes) (**Table 1**). Pathogenetic findings suggested that the majority of microorganisms detected are bacteria, regardless of whether it is mNGS or culture. Fungi detected in culture are only from samples collected through catheters, and mNGS results showed that more viruses are detected in samples collected through catheters. In our study, no virus culture was performed (**Supplementary Figure 1A**). Urine samples tested using mNGS, whether within the range of bacteria and fungi or all pathogens (bacterial, fungal, viral), no negative cases were found in any of the transcatheter-collected urine samples, and the proportion of polymicrobial samples was greater (**Supplementary Figures 1B, C**).

### Consistency of mNGS with conventional culture

**Figure 4:**
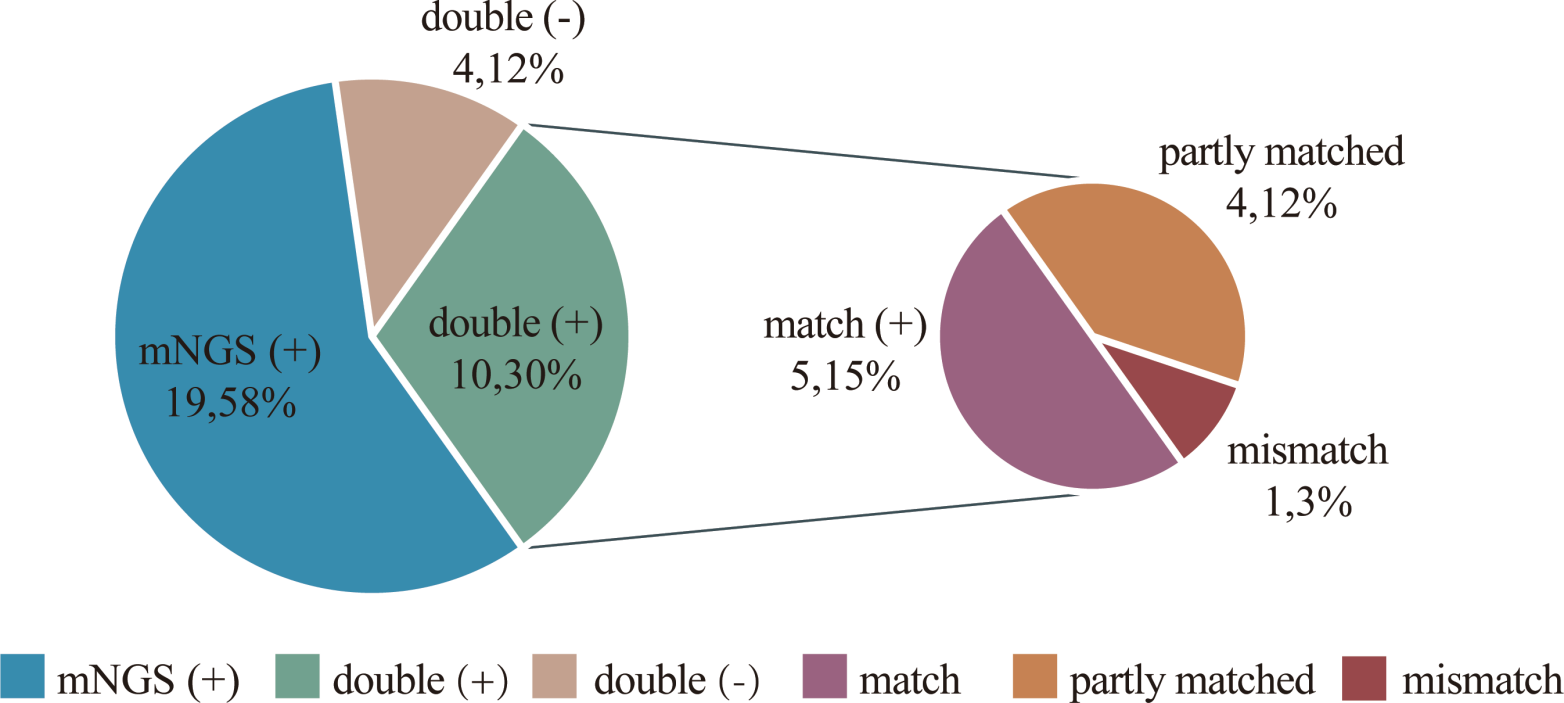
Concordance between mNGS and culture in detection of pathogen. The pie chart demonstrating the positivity distribution for detection of pathogen by two tests in 33 cases. mNGS is more advantageous in detecting pathogens. 19 (58%) cases had pathogens detected by mNGS only. For the double-positive subset, 4 cases were found to be partly matched, meaning that at least one pathogen identified in the test was confirmed by the other.

In the detection of bacteria and fungi, 10 (30.3%) cases showed positive for both methods (double positive), 4 (12.1%) cases showed negative for both methods (double negative), while 19 (57.6%) cases had pathogens detected by mNGS only. Notably, there were no cases in which the conventional method showed positive while the mNGS showed negative. Within the 10 double-positive cases, the results of the two tests were compared, and 5 cases were completely matched and 1 was completely mismatched. The remaining 4 cases were found to be partly matched (**Figure 4** and **Supplementary Table 2**). There are very few cases with conflicting results between the two tests, indicating good consistency between them.

### Effectiveness of mNGS detection in cases with negative conventional test

Among the 23 cases with negative conventional test results, only 4 had negative results for mNGS, while 19 had positive results for bacteria and fungi, 6 of which were monomicrobial and 13 were polymicrobial. Among the 19 pathogen-positive patients, 22 species of pathogens were identified by mNGS. In conclusion, mNGS improved the diagnostic efficiency in cases with negative conventional tests (**Supplementary Table 2**).

### Role and performance of the two assays in the detection of single and mixed pathogens

Single pathogens were detected by mNGS in 11 (33.3%) cases and mixed pathogens in 18 (54.5%) cases; urine culture detected single pathogens in 10 (30.3%) cases and failed to detect the presence of mixed pathogens. All of the mixed pathogens were detected by mNGS. (**Table 6** and **Supplementary Table 2**).

**Table 6:** Results obtained in the detection of single and mixed pathogens (n=33).

No. patients (%)	
Positive for mixed pathogens by mNGS	18 (54.5)
Positive for single pathogen by mNGS	11 (33.3)
Negative for pathogen by mNGS	4 (12.1)
Positive for mixed infections by conventional test	0 (0)
Positive for single pathogen by conventional test	10 (30.3)
Negative for pathogen by conventional test	23 (69.7)

### Role in detection of *M. tuberculosis*

A total of 3 patients positive for *M. tuberculosis* were detected by mNGS. Two of these patients, who were diagnosed with high-risk bladder cancer, received intravesical immunotherapy with bacillus Calmette-Guérin (BCG, a live attenuated vaccine for *Mycobacterium tuberculosis*) as per guidelines (Redelman-Sidi et al., 2014), with specific sequence numbers of 6 and 9, respectively. The remaining patient showed urinary tuberculosis with a specific sequence number of 383 and a positive X-pert test result. This patient with a high suspicion of urinary tuberculosis infection was later diagnosed in an infectious disease hospital.

### Treatment protocol adjustment

For the 25 patients with a positive clinical diagnosis, the treatment protocol was adjusted according to the mNGS results in 23 (92%) patients, while the remaining 2 patients continued their current treatment because the causative organisms were covered by the original antibiotic regimens. All patients showed significant improvement in their urinary symptoms and urinalysis after targeted anti-infection treatment (**Supplementary Table 3**).

### Clinical outcomes

A total of 25 patients were clinically diagnosed with UTI. All of these patients received further targeted anti-infective treatment based on the results of mNGS and urine cultures. Multiple urinalyses before and after treatment were collected in 23 of these patients (the other two patients were not reexamined after treatment). The mean value of white blood cells in high power field (WBCHPF) counts before and after treatment were taken for comparative analysis. The results showed a significant treatment effect, with a statistically significant decrease in urine WBCHPF counts after treatment in these 23 patients (**Table 7** and **Supplementary Table 3**).

**Table 7:** Change in white blood cells in high power field (WBC/HPF) counts of urinalysis before (pre-WBCHPF) and after (post-WBCHPF) targeted anti-infective treatment.

Patients number	pre-WBC/HPF	post-WBC/HPF	P value
P2	369.89	145.16	
P3	460.31	36.25	
P4	195.62	20.00	
P5	86.41	18.75	
P6	30.85	10.15	
P7	103.60	14.60	
P8	159.67	4.16	
P11	393.14	73.28	
P12	116.10	60.70	
P14	229.44	63.54	
P15	153.73	50.64	
P16	140.62	45.22	
P17	107.45	44.92	
P18	166.55	23.32	
P22	74.05	10.95	
P23	109.41	18.78	
P24	85.44	2.61	
P25	259.18	1.42	
P26	37.08	2.55	
P29	47.65	3.74	
P31	129.70	7.00	
P32	104.14	11.00	
P33	167.20	2.07	
WBC/HPF (median, IQR)	129.7(86.41,195.62)	18.75(4.15,45.21)	< 0.001

WBC, white blood cell; HPF, microscope visual field at 400× magnification, high power field; IQR, Interquartile Range.

Changes in voiding are another aspect of assessing the effectiveness of anti-infective therapy. Of the 25 patients, 18 were reviewed for voiding symptoms before and after anti-infective therapy, except for 5 patients who could not be evaluated because of indwelling urinary catheters and 2 patients who underwent percutaneous nephrostomy. 13 of the 18 patients had received empirical anti-infective therapy, and the other 5 patients did not (**Supplementary Table 3**).

**Figure 5:**
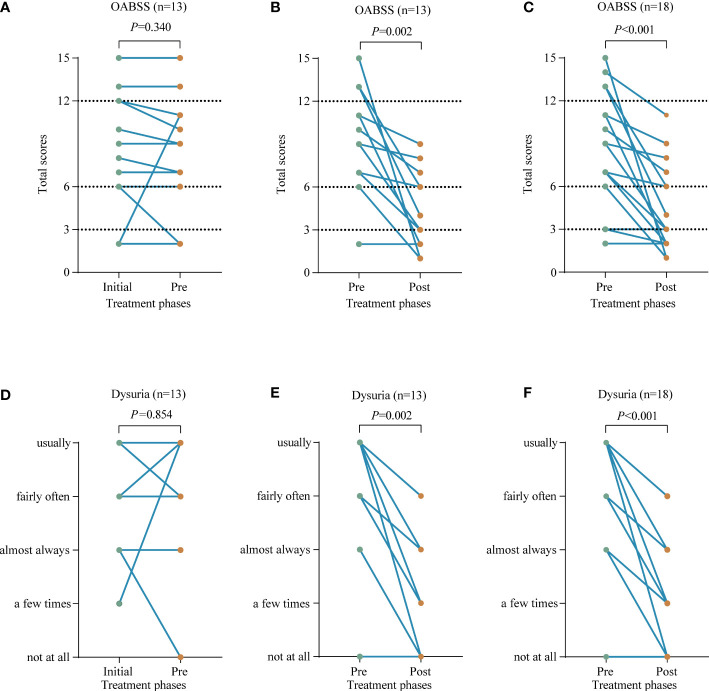
Compare the voiding symptoms before and after receiving anti-infective treatment in terms of overactive bladder syndrome score (OABSS) and dysuria. **(A, D)** The changes in symptom before (Initial) and after (Pre) receiving empirical anti-infective treatment in these 13 patients. **(B, E)** The changes in symptom before (Pre) and after (Post) the above 13 patients were re-treated with targeted anti-infection therapy. **(C, F)** The changes in symptom before (Pre) and after (Post) receiving targeted anti-infective treatment in 18 patients.

The efficacy of the empirical medication was assessed by comparing the voiding symptoms of the 13 patients mentioned above,7/13 patients had no change in overall OABSS; 1/13 patients had no effect of treatment and showed even more severe symptoms; 5/13 patients showed a slight improvement in symptoms. In these 13 patients, the differences in symptom changes were not statistically significant. Among the 5 patients with symptom improvement, 2 patients still had high scores, 10 and 11, respectively, both graded as moderate, tending to be severe (**Figure 5A**). After mNGS detection, these 13 patients received further anti-infective therapy. 11 out of 13 patients had symptom relief after this treatment, and 2 patients had no change in symptom score; no exacerbation of symptoms occurred. The difference before and after this treatment was statistically significant. The 2 patients with no change in symptom scores had a low score of only 2 points before treatment. The patient whose symptom worsened after previous empirical anti-infective treatment had a significant symptom relief, with a decrease in score from 11 to 2 (**Figure 5B**). Changes in voiding symptoms before and after treatment were compared in 18 patients who received targeted anti-infection therapy in the presence of mNGS detection, and the differences were statistically significant (**Figure 5C**). Similarly, empirical anti-infective treatment was ineffective in relieving patients’ dysuria; however, treatments given on the basis of mNGS detection were effective (**Figures 5D–F**).

The median overall OABSS before targeted anti-infective treatment was 9 (IQR: 5-13), and the median daytime frequency, nighttime frequency, urgency and urgency incontinence scores were 1, 3, 4, and 1, respectively. While the median overall OABSS after treatment decreased to 3 (IQR: 2-6), with median scores of 0, 2, 1, and 0 for daytime frequency, nighttime frequency, urgency, and urgency incontinence, respectively (**Figure 5C** and **Supplementary Figure 2**).

## Discussion

Clinically, complicated UTIs are defined as those associated with factors that compromise the urinary tract or host defense, including urinary tract obstruction, immunosuppression, renal failure, and foreign bodies such as indwelling urinary catheters or other drainage devices (Lichtenberger and Hooton, 2008; Levison and Kaye, 2013). In our cases, the majority of patients had complicated UTI that showed recurrent, persistent and refractory features similar to the previous reports(Zhang et al., 2021).

UTI can be caused by Gram-negative and Gram-positive bacteria as well as certain fungi, the most common pathogen being *E. coli* (Foxman, 2014). In complicated UTI, the dominant pathogens are Gram-negative bacilli, of which *E. coli*, *K. pneumoniae* are the most common (Flores-Mireles et al., 2015). The bacteria we detected were mainly Gram-negative bacteria. In our study, the most common pathogen detected by mNGS is also *E. coli*, the enterococcus and anaerobic bacteria also account for a large proportion, especially anaerobes.

UTIs cause major health and economic burden, with current treatment mainly involving culture-based pathogen detection and antibiotic therapy(Tandogdu and Wagenlehner, 2016; Wagenlehner et al., 2020). Methicillin sulfamethoxazole, ciprofloxacin, levofloxacin and ampicillin, are the most common drugs used to treat UTI (Foxman, 2010). However, increasing antibiotic resistance and disease recurrence have further increased the burden on patients and society. Unfortunately, the effectiveness of pathogen detection and drug susceptible testing is further compromised by the low positivity and time-consuming nature of traditional culture method and the early use of broad-spectrum or even prophylactic use of antimicrobial drugs (Frost et al., 2019; Malik and Bhattacharyya, 2019). The presence of fastidious or slow-growing pathogens also limits the sensitivity of culture-based methods (Gu et al., 2019). mNGS technology is changing this dilemma and enabling clinicians to shift from traditional to genetic-based diagnostics.

As a rapid, highly sensitive, and unbiased technology for detecting pathogens, published reports and clinical studies have shown that mNGS has been successfully applied to a variety of sample types, such as cerebrospinal fluid, respiratory secretions, stool, urine, blood and tissues. However, most studies have targeted sterile sites of detection, such as cerebrospinal fluid, alveolar lavage fluid and blood, etc. to detect the presence or absence of pathogens(Gosiewski et al., 2017; Gu et al., 2019; Han et al., 2019). The applications of mNGS on non-sterile materials (e.g., urine) remains are still scarce and controversial. In one of the few recent studies on urine (Zhang et al., 2021; Zhang et al., 2022), a prospective proof-of-concept study by Janes, V. A and colleagues confirmed the feasibility of metagenomic sequencing in detecting of urinary pathogens and drug resistance (Janes et al., 2022). We conducted a mNGS study in patients with suspected UTI and compared it with conventional test in terms of diagnostic performance, pathogenic findings to demonstrate its significance. In addition, we followed up the changes in urinalyses and voiding symptoms to assess the effectiveness of anti-infective treatment.

The mNGS showed high sensitivity and negative predictive values, with great advantages in both diagnostic and exclusionary diagnosis. However, the specificity was low, at 50%. This may be because the specimens mNGS were collected only in cases when UTI was suspected. This makes the number of negative cases present low, and the calculated specificity relatively unreliable. And there are several strategies that can improve the specificity of mNGS results, including: improved specimen collection and processing, better reference databases, improved sequencing methods and improved bioinformatics pipelines, etc.(Alneberg et al., 2014; Dekker and Dulanto Chiang, 2020). In terms of pathogen detection results, mNGS detected a greater number and variety of pathogens. Although there was no statistical difference in the detection of fungi between the two tests, mNGS still showed the advantage of being able to detect more number and more species of fungi. Anaerobes are rarely cultured and isolated because it requires specific inoculation methods and culture equipment (Jin et al., 2020). In our case, all anaerobic bacteria were detected by mNGS, as were all viral and mixed infections. If the antibiotics used in the anti-infection process of the disease are ineffective against other undetected causative organisms, the infection may further worsen and lead to treatment failure because the antibiotics cannot cover them all (Zakhour et al., 2016). The above results indicate that the antibiotic selection based on the result of mNGS detection could be more comprehensive and effective.

We also found a significant decrease in urine WBC counts after targeted anti-infection treatment in the setting of receiving mNGS detection. Besides, patients with persistent symptoms after receiving empirical anti-infective therapy and those who did not receive empirical antibiotics showed significant improvement in both overall OABSS and individual symptoms after targeted anti-infective therapy, showing a favorable outcome. The same was true for the dysuria in patients.

In the management of infectious diseases, the diagnosis of exclusion is as important as the diagnosis of confirmation, and sometimes the former is even more important. In the real world there are patients with persistent urinary irritation symptoms or with chronic abnormal WBC counts in urine. For these patients, the urinary discomfort and sterile pyuria may be due to the following possible reasons: urinary tract obstruction, tumor, foreign bodies, chemical cystitis, and surgical operations, etc. Because of the unbiased nature and high sensitivity of the mNGS detection, its negative results in these cases may help make the diagnosis of the absence of infection. Nor should the results of mNGS be trusted blindly. Possible reasons for false negatives in mNGS results include limitations in the reference database, low numbers of pathogens in the sample, degradation of strains during pretreatment, and difficulties in breaking down hard cell walls of some pathogens(Greninger et al., 2015; Thoendel et al., 2018; Han et al., 2019; Miller et al., 2019).

Cost is an issue for patients to consider. In China, the cost of mNGS detection is around RMB 3,000 per specimen, which is higher than other single tests. This limits its use as a routine test. However, considering the time and financial cost of multiple conventional detections and the high likelihood of false negative results, the advantage of unbiased testing for all possible pathogens at once is still worth considering. The timing of the test we recommended should be in patients with a negative urine culture prior to mNGS who had poor responses to empirical anti-infective treatment and still suspect of a UTI. With the development of sequencing and bioinformatics technologies, the cost of mNGS will be further reduced and increasingly used in the clinic or even become a routine laboratory test.

It is difficult to distinguish pathogenic microorganisms from colonizing and contaminating non-pathogenic microorganisms (Gu et al., 2019). To exclude contaminating bacteria that may come from sampling, transportation, experiments and other processes, the mNGS detections were performed along with negative controls. When the result suggests a positive, the relative abundance, reads number, and pathogen pathogenicity in urinary tract infection were considered, a parameter (confidence level) was given to provide a reference for assessing the likelihood of infection caused by positive microorganisms. Microorganisms with high confidence levels are likely to be pathogenic; while microorganisms with a medium confidence level require clinical judgment in conjunction. The number of specific sequences helps clinicians to determine the content and abundance of pathogens. We should deepen our understanding of the relationship between microorganisms and infection, and correctly interpret and screen the test results, otherwise blindly carrying out treatment based on mNGS reports will inevitably lead to the abuse of antimicrobials. Professional analysis and reasonable interpretation will be worth the extra effort and will greatly facilitate the clinical application of mNGS.

As a colonizing microorganism, virus can also be found in the urine of healthy individuals. It seems to play many previously unrecognized and important roles, either pathogenic or protective (Salabura et al., 2021). We also detected the presence of some viruses, mainly human polyoma virus, human herpes virus. However, these are not considered to be the cause of UTI, because these viruses are usually associated with asymptomatic persistent or latent infections (Divers et al., 2019). Nevertheless, given the complexity of the urinary environment, the exploration of viruses in urine requires more research.

This study also has limitations, such as its limited testing for drug resistance genes, which only identifies known genes and doesn’t consider changes due to mutations, expression changes, or post-translational modifications (Grumaz et al., 2016). No information on drug sensitivity was provided. The absence of a normal urine control is also a shortcoming of the study. Additionally, being a small retrospective study conducted at a single center, it requires further validation through a larger, multicenter prospective study. Besides, our interpretation of the clinical data may be limited by the fact that the diagnosis of UTI differs slightly from what is recommended by clinical guidelines (Gupta et al., 2017). Nevertheless, these differences do not prevent the assessment of the applicability of the mNGS method in this study and may further contribute to the perfection of the guidelines.

Although there are still some barriers, including cost, quality control, and interpretation, etc. Until mNGS becomes a routine clinical test, these barriers will become temporary as the technology rapidly evolves and experience increases. The treatment of infectious diseases is ongoing to an era of precision diagnosis and targeted therapy. Subsequently, the duration of treatment may be another issue to think about next, as it is just as important as the choice of antibiotics.

## Conclusion

mNGS is an emerging detection technology that has shown clear advantages over traditional tests, particularly in the context of mixed infections and UTIs that are difficult to diagnose and treat. mNGS helps to improve the detection of pathogen, guide changes in treatment strategies, and is an effective complement to urine culture. This further facilitates accurate diagnosis and optimal treatment, helping to shift clinical practice from empirical to targeted therapy. As sequencing technology develops and clinical experience is gained, it holds promise to become a common and practical clinical test.

## Data availability statement

The datasets presented in this study can be found in online repositories. The names of the repository/repositories and accession number(s) can be found below: https://www.cncb.ac.cn/, PRJCA013705.

## Ethics statement

Ethical review and approval was not required for the study on human participants in accordance with the local legislation and institutional requirements. The patients/participants provided their written informed consent to participate in this study. Written informed consent was obtained from the individual(s) for the publication of any potentially identifiable images or data included in this article

## Author contributions

Conception and design of study: HH, KJ, HY, and SH. Acquisition and analysis of data: KJ, SH, ZZ, and YL. Analysis and interpretation of bioinformatics data: HY, HH, HL, KJ, HX, and LW. Drafting the manuscript: KJ. Revising the manuscript critically: SH, CS, HH, MT, and HY. All authors contributed to the article and approved the submitted version.
