# Supplementary material for: Enhancing urinary tract infection diagnosis for negative culture patients with metagenomic next-generation sequencing (mNGS)
Source: Front Cell Infect Microbiol. 2023 Mar 3;13:1119020. doi: 10.3389/fcimb.2023.1119020 (PMC10020507; doi:10.3389/fcimb.2023.1119020)
Supplement: Supplementary file 1 [file DataSheet_1.docx]

Supplementary Material

Enhancing Urinary Tract Infection Diagnosis for Negative Culture Patients with Metagenomic Next-Generation Sequencing (mNGS)**Kaipeng Jia^†^, Shiwang Huang^†^, Chong Shen, Hongjun Li, Zhe Zhang, Lei Wang, Gangjian Zhao, Zhouliang Wu, Yuda Lin, Han Xia, Mingze Tang，Huifen Yang*, Hailong Hu***

*** Correspondence:**

Hailong Hu：huhailong@tmu.edu.cn

Huifen Yang：yhsy1122@sina.com

# Supplementary Tables

| **Supplementary Table 1** \| Overactive bladder symptom score (OABSS) | | |
| --- | --- | --- |
| **Question** | **Frequency** | **Score** |
| How many times do you typically urinate from waking in the morning until sleeping at night? | ≤7 | 0 |
|  | 8-14 | 1 |
|  | ≥15 | 2 |
| How many times do you typically wake up to urinate from sleeping at night until waking in the morning? | 0 | 0 |
|  | 1 | 1 |
|  | 2 | 2 |
|  | ≥3 | 3 |
| How often do you have a sudden desire to urinate, which is difficult to defer? | Not at all | 0 |
|  | Less than once a week | 1 |
|  | Once a week or more | 2 |
|  | About once a day | 3 |
|  | 2–4 times a day | 4 |
|  | 5 times a day or more | 5 |
| How often do you leak urine because you cannot defer the sudden desire to urinate? | Not at all | 0 |
|  | Less than once a week | 1 |
|  | Once a week or more | 2 |
|  | About once a day | 3 |
|  | 2–4 times a day | 4 |
|  | 5 times a day or more | 5 |

Patients were instructed to choose the score that best applied to their urinary condition during the past week; the overall score was the sum of the four scores.

**Supplementary Table 2 |** Bacterial and fungal profiles of urinary samples

| **Number of samples** | **Species (urine culture)** | **Species (mNGS)** |
| --- | --- | --- |
| S01 |  |  |
| S02 | *Escherichia coli* | *Escherichia coli* |
| S03 |  | *Pseudomonas aeruginosa*  *Mycobacterium tuberculosis* |
| S04 | *Escherichia coli* | *Escherichia coli* |
| S05 |  | *Enterococcus faecalis*  *Enterococcus faecium* |
| S06 | *Candida* | *Candida*  *Citrobacter freundii* |
| S07 | *Enterococcus faecium* | *Escherichia coli* |
| S08 |  | *Corynebacterium aurimucosum*  *Prevotella bivia*  *Enterococcus faecalis* |
| S09 |  | *Corynebacterium aurimucosum*  *Enterobacter cloacae complex* |
| S10 |  |  |
| S11 |  | *Enterococcus faecalis*  *Talaromyces marneffei* |
| S12 |  | *U.parvum*  *Gardnerella vaginalis* |
| S13 |  | *Corynebacterium singulare* |
| S14 |  | *Mycobacterium tuberculosis* |
| S15 | *Enterococcus faecium* | *Enterococcus faecium* |
| S16 |  | *Corynebacterium aurimucosum* |
| S17 |  | *Prevotella bivia* |
| S18 |  | *Prevotella bivia*  *Escherichia coli* |
| S19 | *Escherichia coli* | *Escherichia coli* |
| S20 | *Escherichia coli* | *Escherichia coli*  *Canidia Albicans* |
| S21 |  |  |
| S22 |  | *Actinotignum schaalii*  *Enterococcus raffinosus*  *Escherichia coli* |
| S23 |  | *Mycoplasma hominis*  *U.urealyticum* |
| S24 |  | *Gardnerella vaginalis*  *Prevotella bivia*  *U.urealyticum*  *Mycoplasma hominis*  *Canidia Albicans* |
| S25 | *Streptococcus agalactiae* | *Streptococcus agalactiae* |
| S26 | *Escherichia coli* | *Escherichia coli*  *Klebsiella pneumoniae* |
| S27 |  | *Enterococcus faecium* |
| S28 |  |  |
| S29 |  | *Corynebacterium urealyticum*  *Prevotella bivia*  *Gardnerella vaginalis*  *Citrobacter freundii*  *Bacteroides fragilis* |
| S30 |  | *Stenotrophomonas maltophilia*  *Acinetobacter baumanii* |
| S31 |  | *Streptococcus agalactiae*  *Escherichia coli* |
| S32 | *Pseudomonas aeruginosa* | *Pseudomonas aeruginosa*  *Aggregatibacter segnis* |
| S33 |  | *Mycobacterium tuberculosis*  *Enterococcus faecalis* |

**Supplementary Table 3** | The results of mNGS and culture, treatment protocol adjustment, and clinical outcomes in patients who were clinically diagnosed with UTI

Note: The **Supplementary Table 3** can be found in the uploaded Excel (It can be found in the uploaded Excel).

# Supplementary Figures

**Supplementary Figure 1.** The bar chart represented the distribution of microorganisms shown by the two assays results in urine samples taken under different modes of urination. 24 patients voided through the urethra (Non-catheter-associated) and 8 patients voided through the catheter (Catheter-associated). (A) Fraction of species (bacteria, fungi, and viruses) in different groups; (B) In the range of bacteria, fungi and viruses, the proportion of polymicrobial, monomicrobial, and negative samples detected by mNGS; (C) In the range of bacteria and fungi, the proportion of polymicrobial, monomicrobial, and negative samples detected by mNGS.**Supplementary Figure 2.** Violin plots illustrating the changes of (A) daytime frequency, (B) nighttime frequency, (C) urgency and (D) urgency incontinence scores of 18 cases. Increased thickness of the violin body represents a greater number of cases and a dot represents a patient. (After correction for multiple testing, p < 0.01 is statistically significant)
