# Supplementary figures and images for: Enhancing urinary tract infection diagnosis for negative culture patients with metagenomic next-generation sequencing (mNGS)
Source: Front Cell Infect Microbiol. 2023 Mar 3;13:1119020. doi: 10.3389/fcimb.2023.1119020 (PMC10020507; doi:10.3389/fcimb.2023.1119020)

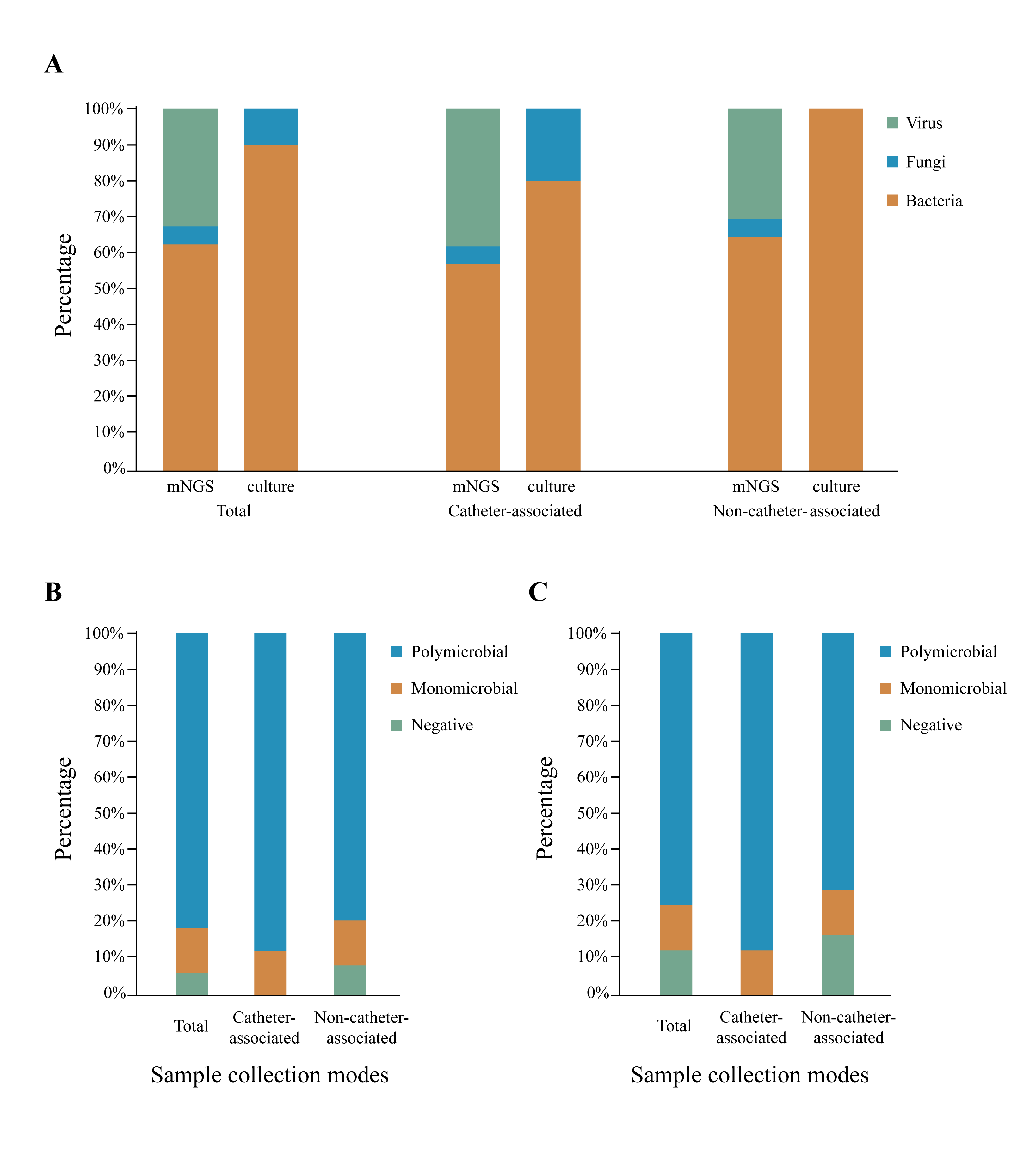

Supplement: Supplementary file 2 [file Image_1.tif]

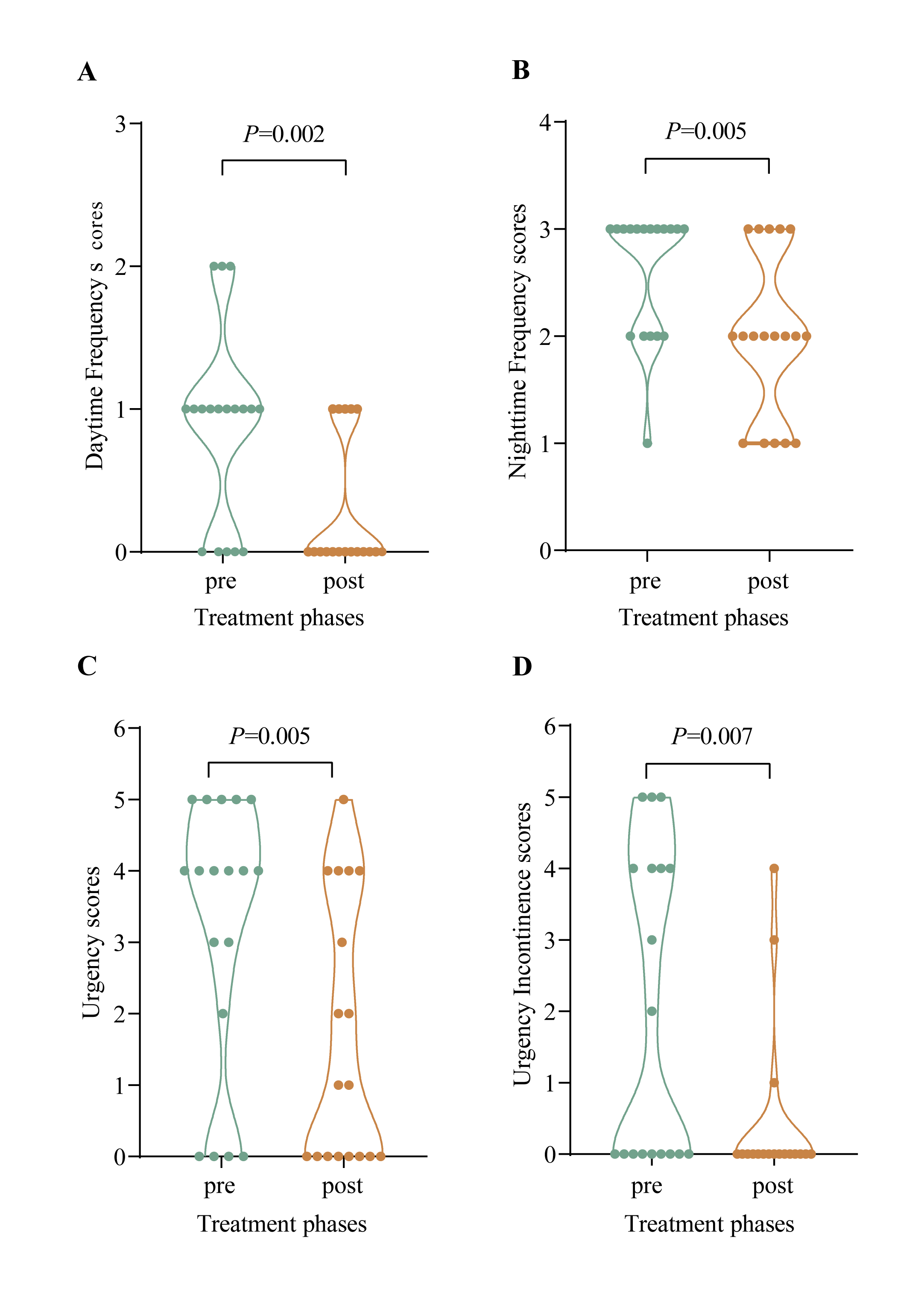

Supplement: Supplementary file 3 [file Image_2.tif]
